# Deliberate Self-Harm Among Youth in the Child Welfare System

**DOI:** 10.1016/j.jaacop.2025.04.002

**Published:** 2025-04-16

**Authors:** Mica Goulbourne, Farah W. Brink, Xueting Xia, Danielle L. Steelesmith, Donna Ruch, Jeffrey A. Bridge, Charmaine B. Lo, Cynthia A. Fontanella

**Affiliations:** a Nationwide Children’s Hospital, Columbus, Ohio; b The Ohio State University College of Medicine, Columbus, Ohio

**Keywords:** children and adolescents, child welfare, deliberate self-harm

## Abstract

**Objective:**

Youth in the child welfare system are at high-risk for self-harm and suicide attempts; yet little is known about factors associated with deliberate self-harm (DSH) following their initial child protective services (CPS) investigation. This study examined factors associated with DSH among youth in the child welfare system.

**Method:**

A retrospective longitudinal cohort analysis was performed using merged data from Ohio Medicaid claims and the Statewide Automated Child Welfare Information Systems for youth ages 5 to 17 with their first CPS investigation between 2010 and 2020 (N = 104,700). Cox proportional hazards analyses were used to examine associations between demographic and clinical factors and DSH within 1 year of the first CPS investigation.

**Results:**

During follow-up, 236 youths experienced a DSH claim. Adolescents (ages 13-17) were more likely to have DSH (odds ratio = 7.51, 99% CI: 5.10-11.06) than young children, with the greatest risk within 15 days of a CPS investigation. There was an increased hazard of DSH for adolescents (vs young children; hazard ratio [HR] = 7.20, 99% CI: 4.88-10.60); girls (vs boys; HR = 2.09, 99% CI: 1.45-3.02); and youth with prior DSH (HR = 26.37, 99% CI: 16.36-42.51), ADHD (HR = 2.59, 99% CI: 1.18-3.77), anxiety (HR = 3.71, 99% CI: 2.58-5.34), depression (HR = 7.38, 99% CI: 5.09-10.70), substance use disorder (HR = 2.66, 99% CI: 1.62-4.36), and thought disorders (HR = 7.14, 99% CI: 2.80-18.22).

**Conclusion:**

Significant risk factors associated with DSH after the first CPS investigation were prior DSH, mental health disorders, female sex, and older youth. Risk of DSH was highest for adolescents in the first 2 weeks of a CPS investigation. Identification of risk factors and high-risk period can inform early intervention to decrease DSH.

## Plain language summary

This study highlights the risk of deliberate self-harm (DSH) among children aged 5 to 17 years involved in child welfare investigations by using data from Ohio Medicaid claims and the Statewide Automated Child Welfare Information Systems (SACWIS). Adolescents aged 13 to 17 years were 7.5 times more likely to harm themselves compared to younger children, particularly within the first two weeks of a Child Protective Services investigation. These findings emphasize the need for early identification of at-risk youth, prompt mental health screenings, and linkage to support services that can help reduce self-harm behaviors.

Youth in the child welfare system are at significantly higher risk for deliberate self-harm (DSH), which includes both nonsuicidal self-injuries (NSSIs) and suicide attempts (SAs), compared with their peers in the general population.^1^, ^2^, ^3^, ^4^, ^5^ A meta-analysis revealed that SAs were 3 times more prevalent among youth in the child welfare system than youth who were not in the system.^4^ Research has also shown that youth who entered the US child welfare system and received out-of-home placement are at increased risk for depression and subsequently suicidal ideation.^1^ A recently published US-based study using data from the National Survey of Child and Adolescent Well-Being (NSCAW) I and II examined the longitudinal patterns of change in suicidal ideation among preteen children (ages 7-12), which identified several risk factors including demographics, child maltreatment, and the presence of mental health symptoms among children engaged in the child welfare system.^2^ As a result of these findings, researchers have urged for heightened awareness and focus on addressing suicidal behavior among youth engaged with the child welfare system.^3^

Prior studies have shown that youth in the child welfare system have an even higher prevalence of suicidality compared with the general population.^6^, ^7^, ^8^ Furthermore, numerous risk factors associated with suicide have been identified among children within the child welfare system, including out-of-home placement, history of mental health disorders, substance use, chronic medical conditions, and increased mental health service utilization in the months before their deaths.^9^^,^^10^ Child maltreatment has also been linked with a greater risk for DSH, which has been described as a negative coping strategy for heightened emotional responses in children and is a strong predictor for future suicide.^11^, ^12^, ^13^ Certain characteristics of child maltreatment associated with increased suicidal risk include the type, frequency, length of time, and severity of abuse.^8^^,^^14^^,^^15^ More specifically, types of child maltreatment associated with elevated risk for suicidal behavior include physical, sexual, and psychological maltreatment.^16^^,^^17^ Other factors associated with DSH among youth in the child welfare system include frequent changes in foster placements and living in a group home or detention center.^18^^,^^19^

Although substantial literature exists on factors associated with DSH among youth in the child welfare system, notable gaps persist. Primarily, most existing studies are cross-sectional, providing a snapshot rather than a comprehensive understanding of how DSH behaviors progress over time. There is a dearth of longitudinal studies that track youth within the child welfare system, hindering comprehension of the development and continuity of DSH behaviors. Moreover, most studies focus on assessing DSH factors in adolescents and do not include younger children, ignoring age-related cognitive, behavioral, and biological differences.^11^^,^^20^^,^^21^ Additionally, scant information is available regarding DSH risk factors following the initial child welfare case and the periods when DSH risk is at its peak.^2^ These gaps underscore the need for further research to understand factors associated with DSH within this vulnerable population across various developmental stages and time frames.

The present study seeks to address these gaps by examining factors associated with DSH among youth in the child welfare system following their first child protective services (CPS) investigation. In addition, we compare DSH in young children and adolescents based on their identified risks to assess developmental differences. Based on prior research, we hypothesized that DSH would be associated with older age, female sex, prior history of DSH, psychiatric diagnoses, and substantiated abuse and neglect. Understanding risk factors associated with DSH for youth involved with the child welfare system and identifying periods of high risk will allow for more timely identification of youth at risk and increase the early implementation of effective interventions and suicide prevention strategies.

## Method

### Study Design and Cohort

A retrospective longitudinal cohort design was used to examine the association between multiple characteristics and DSH among youth in the child welfare system. All youth 5 to 17 years of age who had their first child welfare case opened in Ohio’s Statewide Automated Child Welfare Information Systems (SACWIS) between July 1, 2010, and December 30, 2020, were identified. DSH was defined using *ICD-9-CM* and *ICD-10-CM* codes that were inclusive of both NSSI and SAs (see Table S1, available online). DSH events that led to medical care were captured in this study. A CPS investigation in Ohio was defined as any child welfare involvement to assess for suspected abuse, neglect, or dependency or whether a family needed services through an alternative response.^22^ All youth with no prior CPS investigations within SACWIS were identified. SACWIS data were merged with Ohio Medicaid Claims data using a previously established algorithm to identify youth continuously enrolled in Medicaid during the 180 days before their first CPS investigation (pre-period) and at least 1 day after the CPS investigation was initiated.^23^^,^^24^ All youth were followed from the day of the CPS investigation until the study outcome (ie, DSH), death, Medicaid disenrollment, 1 year, or December 31, 2020, whichever came first. The final study cohort included 104,700 youths and was dichotomized into young children ages 5 to 12 years (n = 74,748) and adolescents ages 13 to 17 years (n = 29,952). The Institutional Review Board at the investigators’ local institution approved this study.

### Data Sources

Data were extracted from SACWIS and Ohio Medicaid claims. SACWIS data were obtained from the state’s Department of Job and Family Services and included information on dates of CPS investigations and foster care placements, reason for CPS investigation, reason for removal from home, and type of placement setting. Medicaid claims data were obtained from the state’s Department of Mental Health Services and included service use dates, type of services (eg, outpatient, inpatient, emergency department), diagnosis codes, and procedure codes, as well as demographic and eligibility information. Medicaid claims data were also linked with death certificate data using a matching algorithm from prior research to identify end of follow-up.^23^^,^^24^ Data used for analyses are protected under a data use agreement and not publicly available. The code used for analyses can be obtained from the corresponding author upon request.

### Outcome Measure

The primary outcome of interest was time to first DSH claim during the follow-up period (see Table S1 for codes, available online). Everyone in the cohort either had a DSH claim or was censored. Time was measured in days from the initiation of the CPS investigation to the first DSH claim or censoring and could range from 1 to 365 days.

### Demographic, Child Welfare, and Clinical Characteristics

Demographic variables included age at date of CPS investigation (5-12 years, 13-17 years), sex, and self-identified race/ethnicity (non-Hispanic White, non-Hispanic Black, Hispanic, other: Asian, American Indian/Alaskan Native, Native Hawaiian or other Pacific Islander, multiple races, unknown race). Child welfare characteristics included any involvement after the first CPS investigation, any removals from the home (placements), and whether claims of abuse were substantiated. CPS investigations after the first investigation and placements were treated as time-varying covariates that started at 0 and increased to 1 if at least 1 additional CPS investigation was initiated or the child was ever removed from home.

Clinical characteristics included a history of DSH, mental health diagnoses, and medical conditions. Mental health diagnoses included anxiety disorders, attention-deficit/hyperactivity disorder (ADHD), depressive disorders, conduct disorders including oppositional defiant disorder, substance use disorders, and thought disorders (Table S2, available online). The grouping for mental health disorders was based on the Hierarchical Taxonomy Of Psychopathology (HiTOP) Primer for Mental Health Researchers.^25^ Pediatric medical conditions were classified as complex chronic, noncomplex chronic, and nonchronic based on the Pediatric Medical Comorbidity Algorithm.^23^^,^^24^^,^^26^ Classifications are determined based on the number of body systems involved and whether or not the conditions are progressive or malignant. History of DSH and the medical conditions were identified during the pre-period. All mental health diagnoses were treated as time-varying covariates, where individuals who had the condition during the pre-period were considered to have the condition during all of follow-up and individuals who did not have the condition during the pre-period started follow-up without the condition and then were changed to having the condition when they received the diagnosis via a Medicaid claim.

### Statistical Analysis

Sample characteristics were examined overall and by age group using percentages. Differences in demographic and clinical characteristics between the 2 age groups were compared using logistic regression with the younger group as the reference category. Unadjusted rates of DSH were calculated for all demographic and clinical characteristics using the total DSH claims per overall person-years for each characteristic. Cox proportional hazard regression models were used to examine associations between demographic and clinical characteristics and DSH. Variation by age was examined using additional Cox proportional hazard models with an interaction term between age and the characteristic of interest while adjusting for sex and race/ethnicity. Hazard ratios (HRs) and associated 99% CIs were calculated for all models, and cumulative incidence functions were plotted. Statistical analyses were conducted using SAS Enterprise Guide 8.3 (SAS Institute Inc, Cary, NC) and R 4.3.0 (R Foundation for Statistical Computing, Vienna, Austria).

## Results

Table 1 summarizes the demographic, child welfare, and clinical characteristics of the cohort and the differences between young children and adolescents across all characteristics. At the time of the first CPS investigation, 71.4% of the study cohort were 5 to 12 years old, 50.6% were girls, and 60.6% were non-Hispanic White race/ethnicity. Most youth (99.7%) were followed for the entire 1-year follow-up period, and 236 (0.2%) had a DSH claim within a year of the first CPS investigation. Adolescents were more likely to have a DSH claim than young children (odds ratio [OR] = 7.53, 99% CI: 5.11-11.09). They also had higher odds of an out-of-home placement (OR = 2.02, 99% CI: 1.82-2.23), substantiated abuse (OR = 1.12, 99% CI: 1.07-1.17), and prior DSH (OR = 12.40, 99% CI: 8.50-18.10) and lower odds of additional CPS investigations after the first investigation (OR = 0.94, 99% CI: 0.90-0.99) compared with young children. For psychiatric comorbidities, adolescents had higher odds of anxiety disorders (OR = 2.36, 99% CI: 2.24-2.48), depression (OR = 6.06, 99% CI: 5.70-6.44), conduct disorders/oppositional defiant disorder (OR = 1.33, 99% CI: 1.23-1.43), substance use (OR = 32.43, 99% CI: 27.46-38.30), and thought disorders (OR = 5.00, 99% CI: 4.19-5.95) and lower odds of ADHD (OR = 0.86, 99% CI: 0.82-0.90) than young children.

**Table 1:** Demographic and Clinical Characteristics of Young Children and Adolescents in Child Welfare System and Ohio Medicaid With First Child Protective Services (CPS) Investigation From 2010 to 2020

Characteristics	Total sample (N = 104,700)	Young children (5-12 y) (n = 74,748)	Adolescents (13-17 y) (n = 29,952)	OR of adolescents group	99% CI
	%	%	%		
**Demographic characteristics**					
Sex					
Male	49.4	50.9	45.4	1.00	Reference
Female	50.6	49.1	54.6	**1.25**	**1.20-1.29**
Race/ethnicity					
Hispanic	5.1	5.1	4.9	**0.96**	**0.88-1.04**
Non-Hispanic Black	28.0	28.5	26.9	**0.94**	**0.90-0.98**
Non-Hispanic White	60.6	60.5	61.0	1.00	Reference
Other^a^	6.3	5.9	7.1	**1.19**	**1.11-1.28**
Additional investigations after first CPS investigation	18.5	18.7	17.9	**0.94**	**0.90-0.99**
Any placements	2.6	2.0	4.0	**2.02**	**1.82-2.23**
Any substantiated abuse	20.0	19.4	21.3	**1.12**	**1.07-1.17**
**Clinical characteristics**					
DSH	0.2	0.1	0.6	**7.53**	**5.11-11.09**
Prior DSH	0.3	0.1	0.9	**12.40**	**8.50-18.10**
Psychiatric comorbidities					
ADHD	17.8	18.4	16.2	**0.86**	**0.82-0.90**
Anxiety	10.7	8.1	17.2	**2.36**	**2.24-2.48**
Depression	8.7	4.0	20.3	**6.06**	**5.70-6.44**
ODD/conduct disorder	5.5	5.0	6.6	**1.33**	**1.23-1.43**
Substance use disorder	3.2	0.4	10.3	**32.43**	**27.46-38.30**
Thought	0.9	0.4	2.2	**5.00**	**4.19-5.95**
Pediatric medical disorders					
No chronic medical condition	81.5	82.1	80.0	1.00	Reference
Noncomplex, chronic medical condition	14.1	13.9	14.4	**1.06**	**1.01-1.11**
Complex, chronic medical condition	4.4	4.0	5.6	**1.45**	**1.34-1.57**

Note: Reference groups are male, Non-Hispanic White, and no chronic medical condition. Boldface type indicates significance under 99% confidence level. ADHD = attention-deficit/hyperactivity disorder; DSH= deliberate self-harm; ODD = oppositional defiant disorder; OR = odds ratio.

a Other race includes Asian Americans, Native Americans/Alaska Natives, Native Hawaiians or other Pacific Islanders, multirace, and unknown.

**Figure 1:**
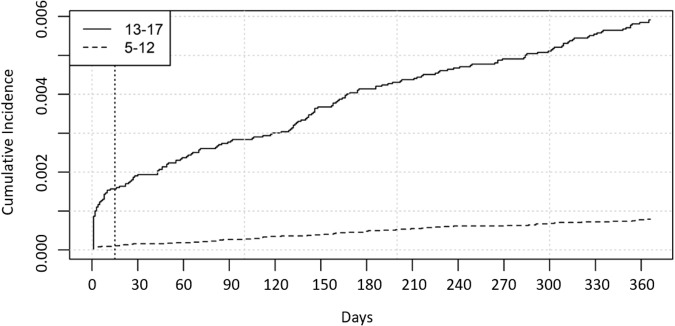
Cumulative Incidence of Deliberate Self-Harm for Young Children and Adolescents During the 365 Days Following a Child Protective Services Investigation

Table 2 shows the rates of DSH and the Cox proportional hazard model results for the entire cohort. The unadjusted DSH rate was 2.3 per 1,000 person-years for all youth, 0.8 per 1,000 person-years for young children, and 5.5 per 1,000 person-years for adolescents. For youth with prior DSH, the unadjusted DSH rate was 123.7 per 1,000 person-years. After adjusting for age, sex, and race/ethnicity, the hazard of a DSH claim was greater for adolescents ages 13 to 17 years compared with young children ages 5 to 12 years (HR = 7.20, 99% CI: 4.88-10.60); girls compared with boys (HR = 2.09, 99% CI: 1.45-3.02); youth with prior DSH (HR = 26.37, 99% CI: 16.36-42.51); and youth with diagnoses of ADHD (HR = 2.59, 99% CI: 1.18-3.77), anxiety (HR = 3.71, 99% CI: 2.58-5.34), depression (HR = 7.38, 99% CI: 5.09-10.70), substance use disorder (HR = 2.66, 99% CI: 1.62-4.36), and thought disorders (HR = 7.14, 99% CI: 2.80-18.22). Figure 1 depicts the cumulative incidence curve for DSH separately for young children and adolescents. The adolescent curve shows a steep increase in hazard within the first 15 days of the CPS investigation and then increases at a lower, steady rate thereafter. For young children, the curve maintains a more constant hazard across the year after the first CPS investigation with lower hazard of DSH than the adolescent group.

**Table 2:** Deliberate Self-Harm (DSH) in Youth in Child Welfare System With First Child Protective Services (CPS) Investigation From 2010 to 2020

Characteristics	No. youth	Person-years at risk	No. first DSH events	Unadjusted DSH rate (per 1,000 person-years)	Unadjusted HR of DSH	99% CI	Adjusted HR of DSH^a^	99% CI
Total	104,700	104,612.4	236	2.3	—	—	—	—
**Demographic characteristics**								
Age at index date, y								
5-12	74,748	74,763.5	59	0.8	1.00	Reference	1.00	Reference
13-17	29,952	29,848.9	164	5.5	**7.51**	**5.10-11.06**	**7.20**	**4.88-10.60**
Sex								
Male	51,671	51,656.2	66	1.3	1.00	Reference	1.00	Reference
Female	53,029	52,956.2	157	3.0	**2.32**	**1.59-3.38**	**2.09**	**1.45-3.02**
Race/ethnicity								
Hispanic	5,314	5,309.0	10	1.9	0.82	0.35-0.90	0.82	0.36-1.91
Non-Hispanic Black	29,348	29,329.2	58	2.0	0.89	0.60-1.32	0.92	0.62-1.37
Non-Hispanic White	63,481	63,427.0	135	2.1	1.00	Reference	1.00	Reference
Other^b^	6,557	6,216.0	20	3.2	1.33	0.72-2.45	1.23	0.66-2.27
Additional investigations after first CPS investigation	19,341	19,344.3	25	1.3	1.68	0.97-2.92	1.68	0.97-2.92
Any placements	2,727	2,726.2	26	9.5	2.12	0.73-6.19	1.71	0.59-4.99
Any substantiated abuse	20,888	20,875.3	38	1.8	0.82	0.52-1.28	0.75	0.48-1.18
**Clinical characteristics**								
Prior DSH	332	299.1	37	123.7	**64.09**	**40.41-101.65**	**26.37**	**16.36-42.51**
Psychiatric comorbidities								
ADHD	18,633	18,613.7	52	2.8	**2.12**	**1.46-3.06**	**2.59**	**1.18-3.77**
Anxiety	11,207	11,160.2	80	7.2	**5.89**	**4.14-8.37**	**3.71**	**2.58-5.34**
Depression	9,120	9,030.2	141	15.6	**14.06**	**10.03-19.71**	**7.38**	**5.09-10.70**
ODD/conduct disorder	5,725	5,724.5	9	1.6	1.22	0.61-2.43	1.18	0.59-2.35
Substance use disorder	3,341	3,321.3	35	10.5	**6.34**	**3.92-10.24**	**2.66**	**1.62-4.36**
Thought	982	972.3	14	14.4	**13.80**	**5.42-35.11**	**7.14**	**2.80-18.22**
Pediatric medical disorders								
No chronic medical condition	85,355	85,301.3	176	2.1	1.00	Reference	1.00	Reference
Noncomplex, chronic medical condition	14,717	14,697.2	36	2.4	1.22	0.77-1.92	1.19	0.76-1.87
Complex, chronic medical condition	4,628	4,613.9	11	2.4	1.09	0.49-2.43	0.93	0.42-2.08

Note: Reference groups are children age 5-12, male, Non-Hispanic White, and no chronic medical condition. Boldface type indicates significance under 99% confidence level. ADHD = attention-deficit/hyperactive disorder; HR = hazard ratio; ODD = oppositional defiant disorder.

a Models are adjusted for age, sex, and race/ethnicity.

b Other race includes Asian Americans, Native Americans/Alaska Natives, Native Hawaiians or other Pacific Islanders, multirace, and unknown.

Table 3 presents the differences in risk factors for young children and adolescents based on interaction terms in the Cox proportional hazard models. The significant risk factors for both age groups are female sex, prior DSH, ADHD, anxiety, depression, and thought disorders. Among those risk factors, prior DSH (*p* = .001), anxiety (*p* < .001), and depression (*p* = .002) have significant interactions between age groups. Substance use disorders have increased hazard for adolescents (HR = 2.60, 99% CI: 1.58-4.23), but no significant results were shown for young children.

**Table 3:** Hazard Ratios for Deliberate Self-Harm (DSH) in Youth in Child Welfare System With First Child Protective Services (CPS) Investigation From 2010 to 2020, Stratified by Age

Characteristics	Young children (5-12 y) adjusted HR^a^	99% CI	Adolescents (13-17 y) adjusted HR^a^	99% CI	Interaction *P*
**Demographic characteristics**					
Sex					.61
Male	1.00	Reference	1.00	Reference	
Female	**2.37**	**1.14-4.90**	**2.00**	**1.31-3.07**	
Race/ethnicity					.35
Hispanic	1.19	0.31-4.60	0.68	0.23-2.01	
Non-Hispanic Black	0.60	0.39-4.48	1.05	0.67-1.63	
Non-Hispanic White	1.00	Reference	1.00	Reference	
Other^b^	1.32	0.39-4.48	1.20	0.59-2.45	
Additional investigations after first CPS investigation	1.74	0.61-4.98	1.67	0.89-3.13	.92
Any placements	1.74	0.13-23.48	1.71	0.53-5.51	.99
Any substantiated abuse	0.55	0.19-1.54	0.82	0.50-1.35	.36
**Clinical characteristics**					
Prior DSH	**118.68**	**35.46-397.22**	**22.95**	**13.81-38.13**	**.001**
Psychiatric comorbidities					
ADHD	**3.15**	**1.54-6.43**	**2.42**	**1.57-3.75**	.42
Anxiety	**8.98**	**4.48-18.02**	**2.89**	**1.91-4.36**	**<.001**
Depression	**15.90**	**1.78-142.06**	**2.98**	**4.00-8.93**	**.002**
ODD/conduct disorder	1.23	0.27-5.68	1.17	0.54-2.53	.93
Substance use disorder	6.78	0.50-91.20	**2.60**	**1.58-4.23**	.35
Thought	**17.22**	**1.28-232.19**	**6.57**	**2.42-17.86**	.37
Pediatric medical disorders					.63
No chronic medical condition	1.00	Reference	1.00	Reference	
Noncomplex, chronic medical condition	0.99	0.37-2.65	0.44	0.03-5.91	
Complex, chronic medical condition	1.25	0.75-2.10	1.06	0.46-2.46	

Note: Reference groups are male, Non-Hispanic White, and no chronic medical condition. Boldface type indicates significance under 99% confidence level. ADHD = attention-deficit/hyperactive disorder; HR = hazard ratio; ODD = oppositional defiant disorder.

a Models were adjusted for sex and race/ethnicity.

b Other race includes Asian Americans, Native Americans/Alaska Natives, Native Hawaiians or other Pacific Islanders, multi-race, and unknown.

## Discussion

In this population-based sample of 104,700 youths with Medicaid enrollment and child welfare involvement, 0.2% engaged in DSH with the highest risk period occurring within the first 15 days following the initiation of a CPS investigation. In the multivariable analysis, several demographic and clinical risk factors were associated with DSH including female sex; adolescent age group; prior DSH; and psychiatric comorbidities such as anxiety, depression, ADHD, substance use, and thought disorders. It is imperative to highlight that there are multiple stressors within the child welfare system that can put children at risk for DSH. Children may experience difficulty with transitions due to numerous placements, baseline stress associated with being removed from their biological family, feeling isolated due to a lack of trust in caregivers or parental support, being exposed to child maltreatment, and having unmet behavioral health needs.^8^^,^^16^^,^^17^^,^^27^^,^^28^

Female participants in our study population had twice the risk for DSH following the initiation of a CPS investigation than their male counterparts. Similar data from the Web-based Injury Statistics Query and Reporting System (WISQARS) Fatal and Nonfatal Injury Reports showed that emergency department visit rates for DSH among young people 10 to 24 years of age were 2.5 times as high among girls and young women compared with boys and young men (514.4 vs 200.5 per 100,000).^29^^,^^30^ A meta-analysis looking at community-based studies involving adolescents found that the lifetime prevalence of DSH was 16.9%, and girls were more likely to engage in this behavior with a risk ratio of 1.72.^31^ Another cross-sectional survey conducted among adolescents in schools in England found that DSH was approximately 4 times more common in girls than in boys.^32^ The study further found that several factors associated with DSH in this population also varied based on sex. Girls were more likely to engage in DSH due to recent DSH in peers and family, substance use, anxiety, depression, and low self-esteem. However, fewer risk factors were identified for boys, and boys were likely to engage in DSH due to suicidal behavior in friends and family members, substance use, and low self-esteem. Additionally, a retrospective study conducted among adolescents in Singapore found that girls were 3 times more likely to engage in DSH compared with boys.^30^ Overall, our study findings are consistent with findings in most prior studies of significant sex differences in DSH with a higher prevalence in female participants.

In our study, adolescents had a higher risk of engaging in DSH following a CPS investigation compared with young children. This pattern has been similarly observed in previous studies: it has been shown that approximately 23% to 40% of adolescents have engaged in NSSI^22^^,^^30^^,^^33^ with 13 years the mean age of onset.^30^^,^^31^ Furthermore, adolescents with a history of DSH are more likely to continue with DSH. One study showed that about 70% of adolescents who engaged in NSSI later had at least 1 SA, with approximately 55% of these adolescents having multiple SAs.^34^ During the adolescent years, neurodevelopmental changes due to puberty are associated with increased susceptibility to emotional dysregulation and impulsivity,^35^ which can be triggered by exposure to stressors such as child maltreatment. Simultaneously, increased access to technology and social media has resulted in greater opportunities for exposure to peer influence among adolescents. Prior research has shown that adolescents who were aware of DSH behaviors among their peers were more likely to engage in similar behaviors.^36^ These factors all play a part in the higher rates of DSH observed among adolescents.

Our study also investigated potential racial and ethnic differences for DSH. We found no significant association between race/ethnicity and DSH in our cohort, which is consistent with some previous studies.^37^, ^38^, ^39^, ^40^ Other studies have found differences in race/ethnicity and DSH associations, but findings remain mixed.^33^^,^^37^^,^^38^^,^^41^ Given the wide variability in findings among the limited studies in the literature, more research investigating racial/ethnic differences in DSH is warranted, especially among youth within the child welfare system.

In contrast to our hypothesis, the presence of substantiated child maltreatment was not associated with DSH. A California population-based study that examined child and adolescent suicide and involvement of CPS found that having a prior allegation of abuse was significantly associated with suicide.^42^ However, the study found that there was no additional risk for children with substantiated cases of maltreatment compared with children who were referred without substantiated maltreatment. Prior research also suggests that child maltreatment is linked to self-injurious behavior and varies by maltreatment type.^14^^,^^43^ Therefore, our study can be further expanded upon by examining specific types of child maltreatment and DSH risk in future studies.

Youth with a prior history of DSH were more likely to engage in DSH after a CPS investigation in our study. Previous research has shown that about 17% of patients with a history of DSH had a subsequent nonfatal DSH event during a 1-year follow up period.^11^ In a British study investigating DSH in children ages 5 to 12, approximately 32% of children had a repeat episode of DSH after their initial presentation to the hospital for DSH. Markedly, 13.5% had a repeat DSH episode within the first year.^44^ The study also found that 53.1% of the cases were attributed to conflicts with their family or friends; 7.5% were due to child maltreatment related to physical, emotional, or sexual abuse; and 6.5% were due to mental health problems. An English multicenter study that examined children ages 10 to 18 found that repetition of DSH occurred in about 27% of participants.^45^ The study also examined risk factors for repeat DSH and found that prior psychiatric treatment and history of DSH were strongly associated with repetition of DSH. Our study results are consistent with previous research that reported DSH is often repeated and is also associated with an increased risk for suicide in the future. This finding underscores the importance of screening for prior DSH exposure.

Our cohort with psychiatric comorbidities, particularly ADHD, anxiety, depression, substance use disorder, and thought disorders, were found to have a higher risk for DSH following a CPS investigation. More notably, the highest risk for DSH was associated with depression and thought disorders. Generally, our findings are consistent with the existing literature that co-occurring behavioral health disorders are associated with an increased risk for self-injurious or suicidal behaviors.^11^^,^^30^^,^^36^ Children in the child welfare system have been treated for ADHD at rates up to 3 times higher than those in the general population.^46^ A study that investigated the relation between DSH, impulsivity, and history of child maltreatment among college students found that participants who reported a history of child maltreatment had higher levels of negative affect, impulsivity, and DSH.^15^ The study suggested that individuals who have been maltreated are more likely to engage in impulsivity, particularly urgency, in an effort to quickly reduce the intense negative emotions they may experience.

Olfson *et al.*^11^ found that several clinical diagnoses including anxiety, mood disorders, substance use disorder, and schizophrenia were related to an increased risk for repeated DSH. Anxiety has been associated with over-reactivity of the amygdala, which can result in a child’s inability to regulate their emotional response to certain situations.^47^ Chronic toxic stress in early childhood may further exacerbate anxiety-related behaviors, which may manifest in externalizing forms such as DSH.^48^ Our study found a strong association between DSH and depression. Youth with child welfare involvement may have more severe depressive symptoms leading to increased risk for engaging in DSH and other suicidal behaviors. A Canadian study showed that adolescents with more severe depressive symptoms experienced a 40% increase in the total number of DSH episodes that occurred in the prior 6 months.^49^ Another strongly associated risk factor identified for adolescents was the presence of substance use. Substance use can lead to decreased inhibition and impairment in judgment, which can contribute to increased likelihood for DSH to occur in youth. Lauw *et al.*^30^ also found that alcohol use was positively associated with DSH with an OR of 3.49. Thought disorders were strongly associated with DSH. This finding is consistent with a longitudinal cohort study that showed individuals with their first episode of psychosis were at increased risk for engaging in DSH and suicide.^50^

Developmental differences were observed among youth and their corresponding risk factors for DSH. Although anxiety was significant for both age groups, young children with anxiety had an even greater risk of DSH following a child welfare investigation than adolescents. This difference may be related to young children being less able to cope with difficult emotions, resulting in externalizing behaviors such as DSH. Although DSH most often occurs in adolescents, these findings underscore the need for health care professionals and child protective agencies to also screen younger children if these specific risk factors for DSH are identified.

Our study showed that substance use disorder had increased the risk of DSH for adolescents, but not young children. The relation between DSH and substance use disorder among adolescents has been well established in the literature.^51^^,^^52^ This connection may be more pronounced in the adolescent years as teens may have more ready access to alcohol, tobacco, marijuana, or other illicit substances compared with younger children.^53^^,^^54^ This risk factor in older children may help guide robust discussions about DSH and substance use in high school–based educational and prevention programs.

The highest risk period for DSH occurred within the 15 days immediately after initiation of a CPS investigation for adolescents. To our knowledge, ours is the first study to have identified this high-risk period following a CPS investigation. Other studies have found increased risk periods for youth with prior DSH or other precipitating factors such as substance misuse, child abuse, and relationship problems, but not specifically related to a child welfare case.^11^^,^^55^ Child welfare reports are investigated for multiple reasons, including acute mental health crisis, and whether or not they are related to an acute or remote concern for child maltreatment, the circumstances surrounding child welfare involvement and subsequent actions can be a trigger for many children. Child welfare involvement typically can result in increased access to services that may be contributing to greater detection for DSH. Our finding provides a more definitive interval during which children are at greatest risk for DSH, which highlights the most opportune time to connect children with the necessary supports.

This high-risk period for DSH has implications to allow for increased research on suicide prevention strategies within this vulnerable population, appropriate management, and timely support that can aid in the reduction of self-injurious behavior. The standardized implementation of early and frequently administered behavioral health screening tools such as the Ask Suicide-Screening Questions (ASQ) and Columbia-Suicide Severity Rating Scale (C-SSRS) for youth in the child welfare system and linkage to interventions geared at suicide prevention strategies such as safety planning should be considered. For youth identified as high risk on the screening, linkage with a behavioral health provider should occur promptly to facilitate close monitoring. This knowledge should also drive the expansion of the behavioral health provider network as well as increased funding and policies supporting mental health services for children with child welfare system involvement.

To the best of our knowledge, this is the first longitudinal study to identify the period of highest risk for DSH in children following a CPS investigation. However, this study is not without limitations. First, the data used were specific to Ohio youth in the child welfare system and Medicaid, which may not be generalizable to youth involved with child welfare in other states or to youth who are involved in child welfare but not enrolled in Medicaid. Second, diagnoses are based on clinical judgment and are not subject to expert validation through standardized diagnostic procedures. Third, claims data do not capture DSH that does not result in medical care, and thus DSH may be underreported. Whereas prior research suggests that suicidal behavior in youth in the child welfare system is higher than in the general population,^6^, ^7^, ^8^ our findings of a low prevalence of DSH in this sample may be an artifact of missing documentation in the Medicaid claims, the limited designated time period, and the young age group of our sample. Fourth, claims data do not distinguish DSH injuries that have suicidal vs nonsuicidal intent, preventing separate analyses of SAs and nonsuicidal self-harm. Finally, data were not available on other important variables that may be associated with DSH such as family history of mental health issues or suicidal behavior, type of maltreatment, unstable living conditions, and precipitating stressors.

In conclusion, youth in the child welfare system are at increased risk for DSH following a CPS investigation. Additionally, the risk of DSH is highest in the immediate days following a CPS investigation among adolescents. Knowledge of these risk factors and high-risk period should drive the development of early screening tools and linkage to interventions geared at targeted suicide prevention strategies to decrease the risk of DSH and future SAs. Future research should include examining specific types of child maltreatment and their association with DSH following a CPS investigation.

## CRediT authorship contribution statement

**Mica Goulbourne:** Writing – review & editing, Writing – original draft, Conceptualization. **Farah W. Brink:** Writing – review & editing, Writing – original draft, Conceptualization. **Xueting Xia:** Writing – review & editing, Writing – original draft, Formal analysis, Conceptualization. **Danielle L. Steelesmith:** Writing – review & editing, Writing – original draft, Formal analysis, Conceptualization. **Donna Ruch:** Writing – review & editing, Writing – original draft, Conceptualization. **Jeffrey A. Bridge:** Writing – review & editing, Writing – original draft, Conceptualization. **Charmaine B. Lo:** Writing – review & editing, Writing – original draft, Conceptualization. **Cynthia A. Fontanella:** Writing – review & editing, Writing – original draft, Formal analysis, Conceptualization.
