# Supplementary material for: Deliberate Self-Harm Among Youth in the Child Welfare System
Source: JAACAP Open. 2025 Apr 16;3(3):506–15. doi: 10.1016/j.jaacop.2025.04.002 (PMC12414319; doi:10.1016/j.jaacop.2025.04.002)
Supplement: Supplemental Tables [file mmc1.docx]

**Table S1:** International Classification of Disease, 9 and 10 Revision, Clinical Modification [ICD-CM 9 and 10] Codes for Deliberate Self-Harm

| **ICD 9 Codes**  Poisoning  Cutting  Other | E950.0-E952.9  E956  E953-E955, E957-E959 |
| --- | --- |
| **ICD 10 Codes**  Poisoning    Cutting  Other | T36.0X2, T36.1X2, T36.2X2, T36.3X2, T36.4X2, T36.5X2, T36.5X4, T36.6X2, T36.7X2, T36.8X2, T36.92X, T37.0X2, T37.1X2, T37.2X2, T37.3X2, T37.4X2, T37.5X2, T37.8X2, T37.92X, T38.0X2, T38.1X2, T38.2X2, T38.3X2, T38.4X2, T38/5X2, T38.6X2, T38.7X2, T38.802, T38.812, T38.892, T38.902, T38.992, T39.012, T39.092, T39.1X2, T39.2X2, T39.312, T39.392, T39.4X2, T39.8X2, T39.92X, T40.0X2, T40.1X2, T40.2X2, T40.3X2, T40.4X2, T40.5X2, T40.602, T40.692, T40.7X2, T40.8X2, T40.902, T40.992, T41.0X2, T41.1X2, T41.202, T41.292, T41.3X2, T41.42X, T41.5X2, T42.0X2, T42.1X2, T42.2X2, T42.3X2, T42.4X2, T42.5X2, T42.6X2, T42.6X4, T42.72X, T42.8X2, T43.012, T43.022, T43.1X2, T43.202, T43.212, T43.222, T43.292, T43.3X2, T43.4X2, T43.502, T43.592, T43.602, T43.612, T43.622, T43.632, T43.692, T43.8X2, T43.92X, T44.0X2, T44.1X2, T44.2X2, T44.3X2, T44.4X2, T44.5X2, T44.6X2, T44.7X2, T44.8X2, T44.902, T44.992, T45.0X2, T45.1X2, T45.2X2, T45.3X2, T45.4X2, T45.512, T45.522, T45.602, T45.612, T45.622, T45.692, T45.7X2, T45.8X2, T45.92X, T46.0X2, T46.1X2, T46.2X2, T46.3X2, T46.4X2, T46.5X2, T46.6X2, T46.7X2, T46.8X2, T46.902, T46.992, T47.0X2, T47.1X2, T47.2X2, T47.3X2, T47.4X2, T47.5X2, T47.6X2, T47.7X2, T47.8X2, T47.92X, T48.0X2, T48.1X2, T48.202, T48.292, T48.3X2, T48.4X2, T48.5X2, T48.6X2, T48.902, T48.992, T49.0X2, T49.1X2, T49.2X2, T49.3X2, T49.4X2, T49.5X2, T49.6X2, T49.7X2, T49.8X2, T49.92X, T50.0X2, T50.1X2, T50.2X2, T50.3X2, T50.4X2, T50.5X2, T50.6X2, T50.7X2, T50.8X2, T50.902, T50.992, T50.A12, T50.A22, T5A.092, T50.B12, T50.B92, T50.Z12, T50.Z92, T51.0X2, T51.1X2, T51.2X2, T51.3X2, T51.8X2, T51.92X, T52.0X2, T52.1X2, T52.2X2, T52.3X2, T52.4X2, T52.8X2, T52.92X, T53.0X2, T53.1X2, T53.2X2, T53.3X2, T53.4X2, T53.5X2, T53.6X2, T53.7X2, T53.92X, T54.0X2, T54.1X2, T54.2X2, T54.3X2, T54.92X, T55.0X2, T55.1X2, T56.0X2, T56.1X2, T56.2X2, T56.3X2, T56.4X2, T56.5X2, T56.6X2, T56.7X2, T56.812, T56.892, T56.92X, T57.0X2, T57.1X2, T57.2X2, T57.3X2, T57.8X2, T57.92X, T58.02X, T58.12X, T58.2X2, T58.8X2, T58.92X, T59.0X2, T59.1X2, T59.2X2, T59.3X2, T59.4X2, T59.5X2, T59.6X2, T59.7X2, T59.812, T59.892, T59.92X, T60.0X2, T60.1X2, T60.2X2, T60.3X2, T60.4X2, T60.8X2, T60.92X, T61.02X, T61.12X, T61.772, T61.782, T61.8X2, T61.92X, T62.0X2, T62.1X2, T62.2X2, T62.8X2, T62.92X, T63.002, T63.012, T63.022, T63.032, T63.042, T63.062, T63.072, T63.082, T63.092, T63.112, T63.122, T63.192, T63.2X2, T63.302, T63.312, T63.322, T63.332, T63.392, T63.412, T63.422, T63.432, T63.442, T63.452, T63.462, T63.482, T63.512, T63.592, T63.612, T63.622, T63.632, T63.692, T63.712, T63.792, T63.812, T63.822, T63.832, T63.892, T63.92X, T64.02X, T64.82X, T65.0X2, T65.1X2, T65.212, T65.222, T65.292, T65.3X2, T65.4X2, T65.5X2, T65.6X2, T65.812, T65.822, T65.832, T65.892, T65.92X  X78.0XX, X78.1XX, X78.2XX, X78.8XX, X78.9XX, X79.XXX  T14.91, T71.112, T71.122, T71.132, T71.152, T71.162, T71.192, T71.222, T71.232, X72.XXX, X73.0XX, X73.1XX, X73.2XX, X73.8XX, X73.9XX,  X74.01X, X74.02X, X74.09X, X74.8XX, X74.9XX, X75.XXX, X76.XXX, X77.0XX, X77.1XX, X77.2XX, X77.3XX, X77.8XX, X77.9XX, X80.XXX, X81.0XX, X81.1XX, X81.8XX, X82.0XX, X82.1XX, X82.2XX, X82.8XX, X83.0XX, X83.1XX, X83.2XX, X83.8XX |

**Table S2:** International Classification of Disease, 9 and 10 Revision, Clinical Modification [ICD-CM 9 and 10] Codes for Psychosis and Psychiatric Comorbidities

| Category | Psychiatric Comorbidities | ICD 9 Codes | ICD 10 Codes |
| --- | --- | --- | --- |
| Externalizing | ADHD | 314 | F90 |
|  | Disruptive Disorders: Oppositional Defiant and Conduct Disorders | 312, 313.81 | F91 |
| Anxiety | Anxiety Disorders | 300.0-300.3, 309.21, 309.81 | F40-F42, F43.1, F44 |
| Depression* | Depressive Disorder | 296.2, 296.3, 296.92, 300.4, 311 | F32, F33, F34.1 |
| Thought* | Schizophrenia | 295, 297, 298 | F20, F22-F29 |
|  | Mood Disorder with Psychotic Features | 296.54, 296.44, 296.34 | F30.2, F31.2, F32.3 |
|  | Paranoid Personality | 301.0 | F60.0 |
|  | Schizotypal Personality | 301.22 | F21 |
| Substance Use | Substance Use Disorder | 291,292, 303-305 | F10-F19 |

*Major depressive disorder with psychotic features (ICD 9: 296.34, ICD 10: F32.3) is included in both depression and thought.
